# Genetic analysis of acute intermittent porphyria caused by novel classical splicing variant in the insertion region of 29-residue specific to human *HMBS* protein

**DOI:** 10.3389/fmolb.2023.1230798

**Published:** 2023-08-10

**Authors:** Lei Liang, Haixia Meng, Haotian Wu, Jianrong Zhao

**Affiliations:** ^1^ Center for Prenatal Diagnosis and Medical Genetics, Affiliated Hospital of Inner Mongolia Medical University, Hohhot, China; ^2^ School of Public Health, Inner Mongolia Medical University, Hohhot, China; ^3^ Department of Nephrology, Affiliated Hospital of Inner Mongolia Medical University, Hohhot, China

**Keywords:** AIP, *HMBS*, splicing variant, molecular dynamics, molecular docking

## Abstract

**Background:** Acute intermittent porphyria (AIP; OMIM#176000) is a genetic disorder that is caused by mutations in the hydroxymethylbilane synthetase (*HMBS*) gene. This gene encodes the third enzyme in the heme biosynthesis pathway. Human *HMBS* (*hHMBS*) contains a 29-residue insert (residues 296-324) at the interface between domains 1 and 3. The function of this insert is currently unknown. In this study, a previously unidentified classical Splicing variant was discovered in the *HMBS* gene of a female AIP patient from China. The variant was validated through comparison with the patient’s husband and daughter.

**Methods:** Peripheral blood samples were obtained from the patient, the patient’s husband, and their daughter. Gene expression was analyzed using whole exon sequencing and Sanger sequencing. To validate alternative splicing, RNA was extracted from the patient’s peripheral blood and reverse transcribed into cDNA. Aberrant splicing caused by variants was predicted using I-TASSER and PyMOL software to simulate protein structures. Finally, molecular dynamics of the proteins were simulated using the AMBER14sb software.

**Results:** The patient and her daughter have a classical Splicing variant c.912 + 1G>C of the *HMBS* gene. This variant was not found in the patient’s husband and has not been previously reported in scientific literature. Analysis of the patient’s peripheral blood transcripts revealed that c.912 + 1G>C retained intron 13 and resulted in an exon 13 skipping. Further analysis through homology modelling and molecular dynamics showed that this variant alters the secondary structure of the *HMBS* protein, leading to functional differences.

**Conclusion:** This research has discovered a new classical Splicing variant c.912 + 1G>C in the *HMBS* gene that has been identified as pathogenic. This finding not only expands the molecular heterogeneity of AIP but also provides crucial information for genetic diagnosis.

## 1 Introduction

The pathogenic gene *HMBS* responsible for AIP is located on chromosome 11q23.3. *HMBS* consists of 14 exons and encodes 361 amino acids (Bustad et al., 2021; Gonzalez-Mosquera and Sonthalia, 2021). AIP is an autosomal dominant genetic disease with low clinical penetrance (Yasuda et al., 2019). The clinical manifestations of AIP are mainly observed in the nervous system and skin (Norman et al., 2001). Various factors such as drug use, fasting, and hormonal changes can contribute to the acute attack of AIP. All of these factors stimulate the activity of 5′-aminolevulinate synthetase 1 (ALAS1), which is the first rate-limiting enzyme in the heme biosynthesis pathway. Liver ALAS1 induction results in the production of multiple intermediates. However, in cases where *HMBS* enzyme activity is absent, the accumulation of neurotoxic porphyrin precursors such as 5’-aminolevulinic acid (ALA) and porphobilinogen (PBG) occurs in the liver. These precursors are then secreted into the plasma and excreted from the body through urine (Floderus et al., 2006; Besur et al., 2015; Naik et al., 2016; Bissell et al., 2017; Duque-Serrano et al., 2018). The incidence rates of AIP differ across regions, with a total rate of approximately 1.5 per 100,000 individuals. Both men and women can be affected, but women account for 70% of cases (Kauppinen and Mustajoki, 1992; Schuurmans et al., 2001; Kauppinen and von und zu Fraunberg, 2002; Fraunberg et al., 2005). Typically, the onset of the disease occurs between the ages of 20 and 40, with children being less susceptible to the disease. Currently, the validation of classical splicing variation poses a significant challenge in genetic diagnosis. The possibility of further genetic diagnosis is contingent upon the ability to evaluate mutations that alter splicing patterns through functional experiments. Transcription experiments represent a viable option for assessing the effects of mutations on splicing mechanisms, provided that patient cells can be obtained. This study aims to evaluate the authenticity and potential consequences of splicing variant c.912 + 1G>C on the normal splicing of *HMBS*. The article discusses the method used to determine the pathogenicity of the splicing variant and introduces a new idea for predicting the effect of *HMBS* variation on protein conformation using molecular dynamics principles.

## 2 Materials and methods

### 2.1 Ethical compliance and patient information

The Prenatal Diagnostic Centre of the Affiliated Hospital of Inner Mongolia Medical University provided genetic counselling to a female patient who had previously received treatment for sudden general paralysis and was diagnosed with AIP, but had not undergone testing for AIP-related genes. The patient’s husband and daughter had a normal phenotype, and both were included in the genetic counselling process. Peripheral blood samples were collected from the family members, and the study was approved by the Ethics Committee of Inner Mongolia Medical University. The patient provided written informed consent to participate in the study.

### 2.2 Whole exome sequencing and sanger sequencing

Genomic DNA extraction was performed on the patient, her husband, and their daughter using the Blood Genomic DNA Extraction Kit (TIANGEN, China). The Hieff NGS®OnePot DNA Library Prep Kit for Illumina^®^ (YEASEN, China) and xGen Exome Research Panel v1.0 (Integrated DNA Technologies, Inc., United States) were utilized to conduct library preparation and exome capture. The captured libraries were subsequently sequenced on a HiSeq2500 (Illumina, California, CA, United States).

The raw data underwent quality assessment through the utilization of FASTQC. Subsequently, the clean reads were aligned to the reference genome (GRCh37/hg19) via BWA software. Following the removal of duplicates and recalibration of base quality, SNP and Indel variants were identified using the GATK pipeline. The identified variants were then annotated utilizing ANNOVAR and filtered based on minor allele frequencies (MAFs) of <0.5% for dbSNP, 1000G, ExAC, gnomAD databases. Additionally, copy number variation analysis was conducted on probe coverage regions utilizing xhmm and clams tools.

The guidelines for interpreting sequence variation data pertain to the genetic variation classification standards and guidelines established by the American College of Medical Genetics and Genomics (ACMG), the ClinGen sequence variation interpretation (SVI), and the guidelines specific to genes and diseases.

To authenticate the variant, we conducted Sanger sequencing on the peripheral blood DNA samples obtained from the patient, the patient’s daughter, and the patient’s husband. The PCR protocol entailed a pre-denaturation step (95°C for 5 min), followed by 35 cycles of denaturation (95°C for 30 s), annealing (60°C for 30 s), elongation (72°C for 30 s), and a final extension (72°C for 5 min). The amplicon was sequenced using the ABI 3730xl Genetic Analyzer (Applied Biosystems, Inc.), and the resulting sequences were compared to reference sequences using CodonCode Aligner.

### 2.3 RT-PCR, PCR and sequencing

To examine the impact of variation on mRNA splicing, the PAXgene Blood RNA Kit (Qiagen#762174) was utilized to isolate total RNA. Subsequently, reverse transcription was conducted with the Hifair®II 1st Strand cDNA Synthesis Kit (YEASEN Biotech Co., Ltd., China). The variant c.912+1G > C is located in intron 13 of *HMBS* (transcript NCBI RefSeq ID: NM_000190.4), which may affect the splicing between exon 13 and exon 14. Primers were designed following standard protocol with sequences as forward, 5′-GTT​CCC​GCA​TCT​GGA​GTT​CA-3′; reverse, 5′-TGT​GCC​CCA​CAA​ACC​AGT​TA-3′. The PCR product for a wildtype is a length of 633bp encompassing nucleotides 49th nucleotide of exon 8 to 191th nucleotide of exon 14. The PCR reaction system consisted of 0.5 µl Taq DNA Polymerase (Thermo Scientific, China), 2.5 µl 10× Taq Buffer, 2 µl MgCl_2_, 0.5 µl dNTP, 1 µl of each primer, 1 µl cDNA template, and 16.5 µl H_2_O in a total volume of 25 µl. The PCR cycling conditions consisted of an initial denaturation step at 94°C for 5 min, followed by 35 cycles of denaturation at 94°C for 30 s, annealing at 58°C for 30 s, and extension at 72°C for 1 min, with an additional extension step of 8 min at 72°C. The successful amplification of the PCR products was verified through 2% agarose gel electrophoresis, after which the products were subjected to direct sequencing using the ABI 3730xl DNA Analyzer.

### 2.4 The evolutionary conservation analysis of amino acid residues and the structure prediction of mutant proteins

Protein sequences from various species were obtained from NCBI and subjected to multiple sequence alignment and conservation analysis using Jalview software. The influence of the variable region was simulated using the I-TASSER server (http://zhanglab.ccmb.med.umich.edu/I-TASSER/).

### 2.5 3-D structure analysis of HMBS

The 3-D structure of the c.912 + 1G>C mutant was generated from the amino acid sequences of the *HMBS* wild-type. AlphaFold v2.3.1 was employed for structure prediction. The full database and the monomer_ptm model were selected for single-chain prediction, resulting in the generation of structure models.

### 2.6 Molecular dynamics

The protein was loaded into the GROMACS module, and hydrogen atoms and NaCl ions were added (Berendsen et al., 1995; Lindahl et al., 2001; Van Der Spoel et al., 2005; Hess et al., 2008) The TIP3P dominant water model was selected and the periodic boundary conditions were set. The workflow of molecular dynamics simulation included four steps: energy minimization, NVT equilibrium, NPT equilibrium and production dynamics simulation. Firstly, the protein heavy atoms were constrained to minimize the energy of water molecules by 5,000 steps; Then, maintaining the constraints, 50000 step NVT ensemble simulation was carried out for the whole system. The temperature was 298K, and the time step was 2 fs; Then 50000 step NPT ensemble simulation was carried out for the whole system, the temperature was 298 k, and the time step was 2 fs; finally, the molecular dynamics simulation of the system was carried out in the NPT ensemble for 100 ns with a time step of 2 fs. For the specific set of conditions applied for MD simulation, the long-range interactions were processed using the PME method, while the van der Waals action was processed using the cut off method with a truncation value of 1.2 nm. The constant temperature was treated using the V-rescale method, and the air pressure was treated using the Berendsen method, maintaining a pressure of 100 MPa.

### 2.7 Molecular docking simulation of HMBS enzyme activity

The present study generated the 3-D structures of both the *HMBS* wild-type and mute-type proteins, with the dipyrromethane (DPM) cofactor molecule located at the center, by utilizing the 3ECR protein in the PDB as a template. The interaction between the PBG small molecule and the protein was analyzed through the GROMACS version 2021.4 software package module. As for the cofactors used in MD simulations, the PBG small molecule was built via MS software and the Charmm36 force field was applied to this molecule via home-made python code. An appropriate amount of Na and Cl ions were added to neutralize the ion systems. The methodology used for doing analysis on the trajectories included converting the output trajectory file (e.g.,.xtc) into a format suitable for analysis, using the “gmx rms” and “gmx rmsf” commands to calculate root mean square deviation (RMSD) and root mean square fluctuation (RMSF) of the selected region, using the “gmx gyrate” command to calculate the radius of gyration. The above description is the molecular docking protocol used in this study.

## 3 Results

### 3.1 Identification of novel HMBS mutation

**FIGURE 1:**
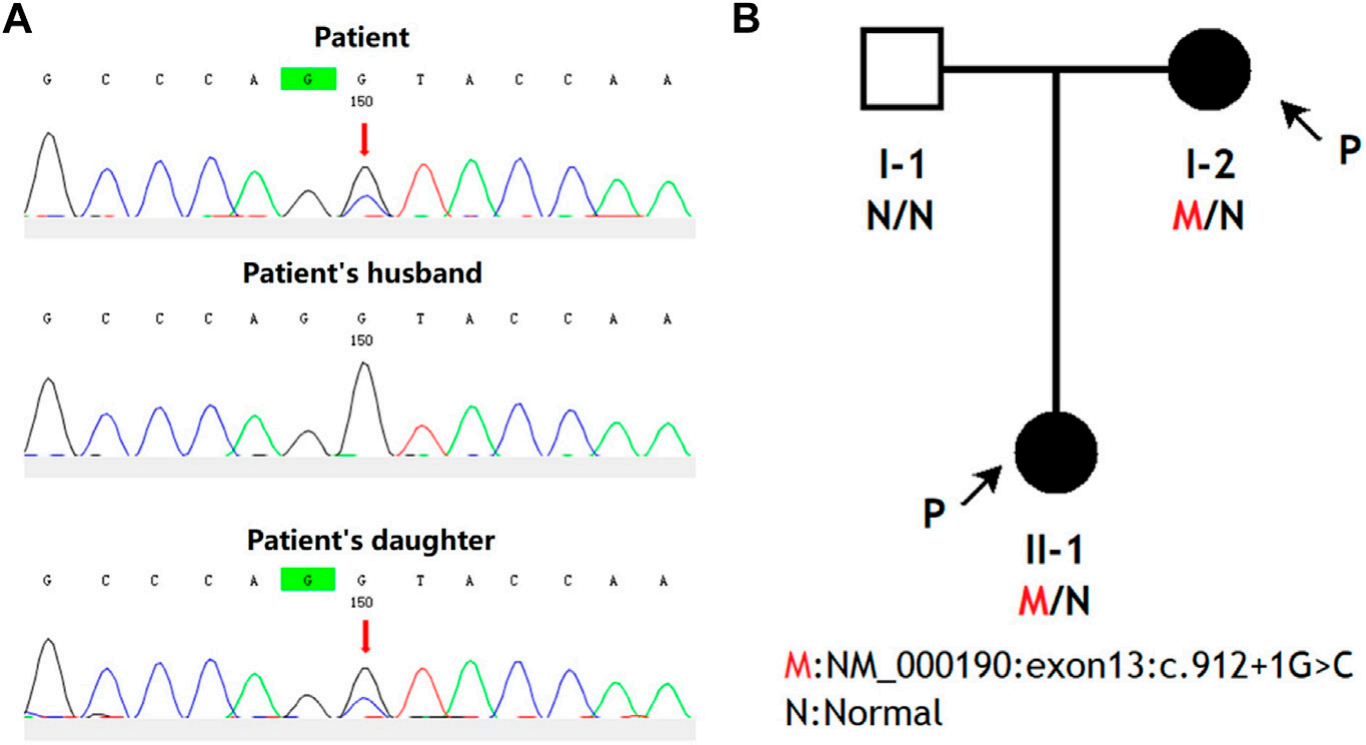
The pedigree diagrams of AIP patients from China and an analysis of *HMBS* gene mutations within this family. **(A)** Interpretation of variation. Sanger sequencing was performed on the genomic DNA samples from the patient, the patient’s daughter, and the patient’s husband. Any genetic variants were denoted by red arrows. **(B)** Family analysis. The black arrows indicate the individuals who participated in this study, namely, the patient and her daughter.

The utilization of whole-exome sequencing led to the identification of a Splicing c.912 + 1G>C heterozygous variant in exon 13 of the *HMBS* gene of the patient, which was subsequently confirmed through Sanger sequencing to be present in her daughter (Figure 1A). The variant was not detected by the patient’s husband, therefore it was inherited by their daughter from the mother (Figure 1B). The variant ID (rs797045752) was absent from both the ExAC and 1000G databases, while the dbSNP database contained the variant ID but lacked information on population frequency. The gnomAD database does not encompass this variant population, and no literature has reported on it.

### 3.2 Corroboration of HMBS mutations as the determinant of alternative splicing in splicing assay

**FIGURE 2:**
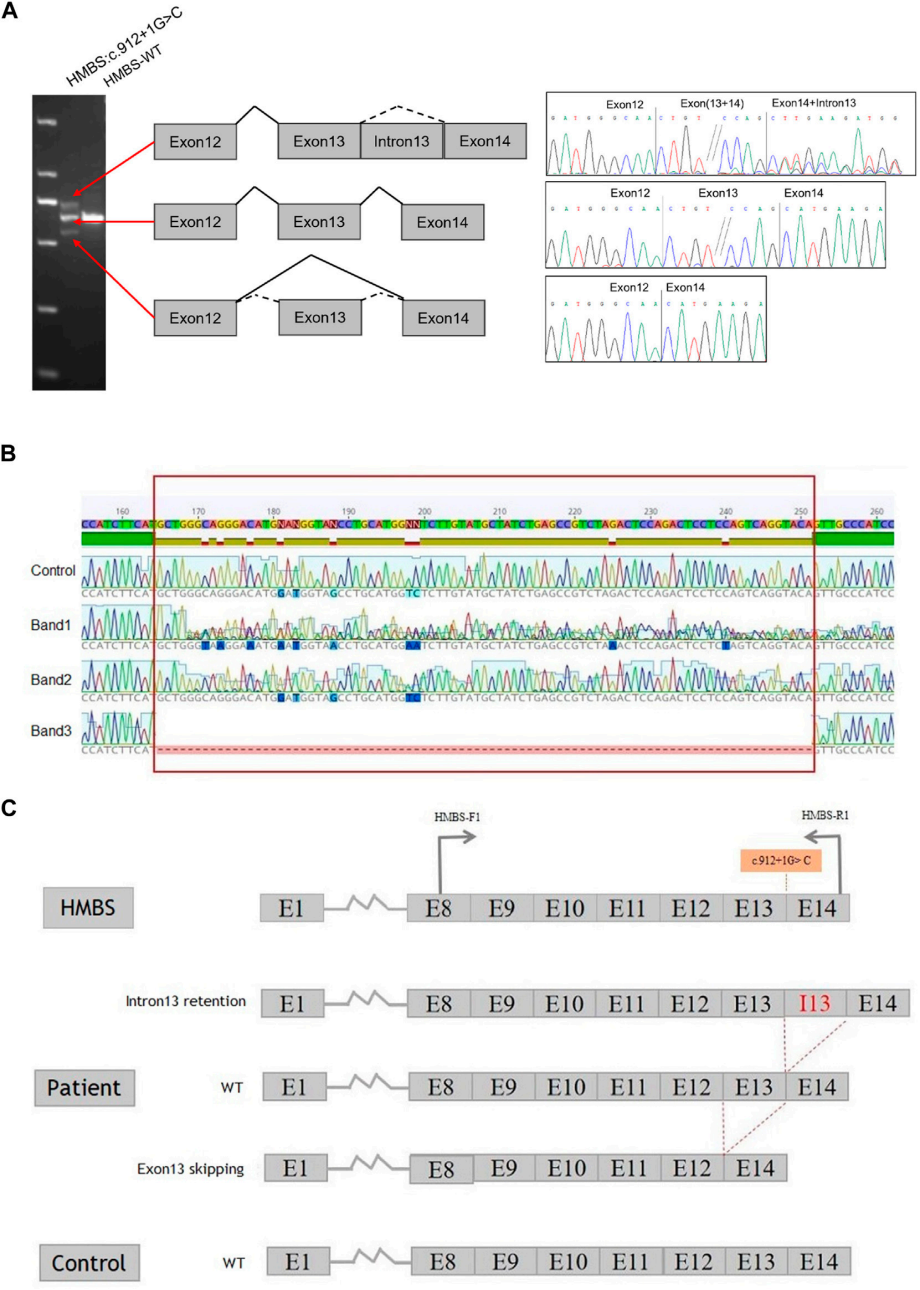
Constructs containing the full length of intron 13 of the *HMBS* gene were utilized to investigate splicing patterns. **(A)** Gene splicing assay. The splicing assay revealed that the amplification product size of the control sample was in accordance with the anticipated amplification length. In comparison to the control sample, the amplification product of the patient’s sample exhibited two additional bands. Specifically, band 1 was identical to the control band, band 2 displayed no variation in size, and band 3 was smaller than the control band, indicating the possibility of anomalous splicing in the cDNA of the patient’s sample. **(B)** *HMBS* gene c.912 + 1G>C transcript validation experiment. The present study employed RT-PCR to examine the RNA in peripheral blood of patient. The control samples exhibited normal sequencing results. However, the sequencing results of band 1 revealed overlapping peaks, suggesting the retention of intron 13 in the cDNA of the sample. The sequencing result of band 2 was identified as wild-type, while the sequencing result of band 3 indicated exon 13 skipping in the cDNA of the sample. It is noteworthy that the region highlighted in red corresponds to exon 13, which spans 87 bp. **(C)** The splicing schematic diagram. The occurrence of c.912 + 1G>C results in both the retention of intron 13 and the skipping of exon 13 in the *HMBS* gene.

Using peripheral blood cDNA as a template, the target region of both patient and normal controls was amplified via PCR with primer *HMBS*-F1/*HMBS*-R1. The amplification product size of the control sample was found to be consistent with the theoretical amplification length. However, in comparison to the control sample, the amplification product from the patient exhibited two additional bands (Figure 2A). The cDNA amplification products from both the patients and control samples were analyzed using Sanger sequencing techniques. Band 1 showed an overlap peak, indicating the retention of intron 13 (by 88 base pairs) in the patient’s cDNA. Band 2 displayed a wild-type sequence, while band 3 revealed that the patient’s samples lacked 87 bp in exon 13, indicating exon 13 skipping (Figure 2B). The sequencing results of the control sample demonstrate that exon skipping and intron retention were not observed, indicating that c.912 + 1G>C had an impact on *HMBS* gene splicing (Figure 2C).

### 3.3 Model analysis of abnormal structure of HMBS caused by mutation

**FIGURE 3:**
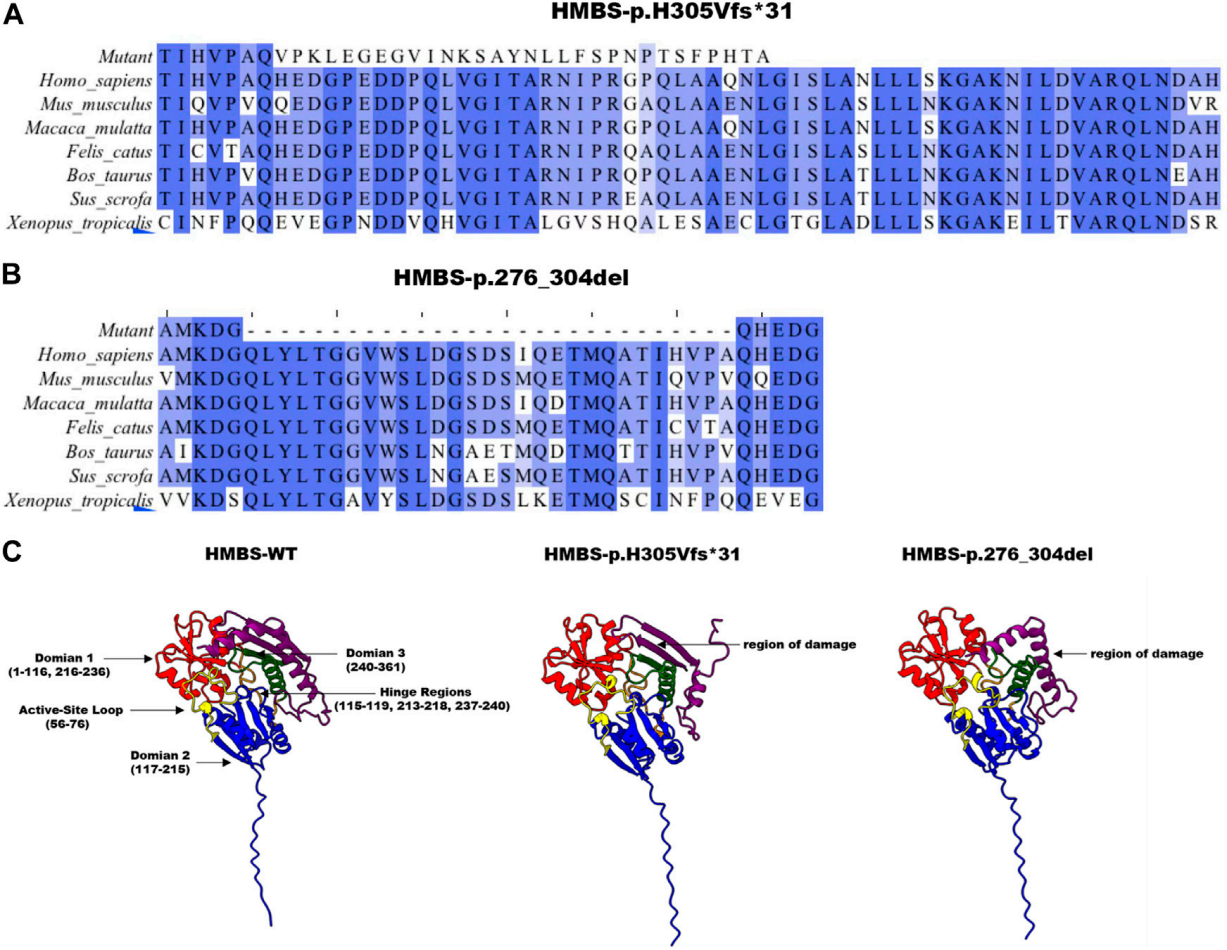
Analysis of *HMBS* mutations. **(A)** The conservation of amino acid residues affected by the c.912 + 1G>C (p.H305Vfs*31) mutation has been observed across various species through the process of evolution. NCBI accession numbers are *Homo sapiens*: NP_000181.2; *Mus musculus*: NP_038579.2; *Macaca mulatta*: NP_001253589.1; *Felis catus*: NP_001171279.1; *Bos taurus*: NP_001039672.1; *Sus scrofa*: NP_001090881.1; *Xenopus tropicalis*: NP_001005635.1. **(B)** The conservation of amino acid residues affected by the c.912 + 1G>C (p.276_304del) mutation has been observed across various species through the process of evolution. Asterisk (−) means missed amino acids. **(C)** The homology modeling analysis was employed to generate ribbon representations of *HMBS*-WT, *HMBS*-p.276_304del, and *HMBS*-p.H305Vfs*31. The amino acid positions of domain 1 were represented in blue and ranged from 1–116 and 216–236. The amino acid positions of domain 2 were represented in red and ranged from 117–215. The amino acid positions of domain 3 were represented in green and ranged from 240–361. The amino acid positions within the hinge region span from 115 to 119, 213 to 218, and 237 to 240, as indicated by the orange coloration. The active site ring amino acid positions, denoted by the yellow coloration, range from 56 to 76. The purple coloration signifies the region of amino acid damage.

Based on the analysis of evolutionary conservation of *HMBS* amino acid residues, it can be inferred that the damaged amino acids exhibit a high degree of conservation across various species (Figures 3A, B). The mutations p.H305Vfs*31 and p.276_304del in the *HMBS* protein result in changes to its Domain3 region. The former causes a shift in the 305th amino acid and early termination of translation, leading to a more than 40% sequence change and shortened α helix in this region, resulting in decreased protein stability. The latter causes amino acid deletions from 276 to 304, leading to more than 20% of the Domain3 region being deleted and a reduced length of α helix in this region, which may also reduce protein stability (Figure 3C). In the absence of molecular dynamics analysis, the conformational changes of p.276_304del and p.H305Vfs*31 in domain 3 can only be simulated through PyMol and modeler homologous modeling in comparison to WT. However, domains 1 and 2 of p.276_304del and p.H305Vfs*31 are not significantly different from those of WT. These results indicate that homologous modeling without molecular dynamics analysis can only simulate local changes caused by the mutation, and not the overall conformational impact of the mutation.

### 3.4 Protein molecular dynamics simulation of HMBS

**FIGURE 4:**
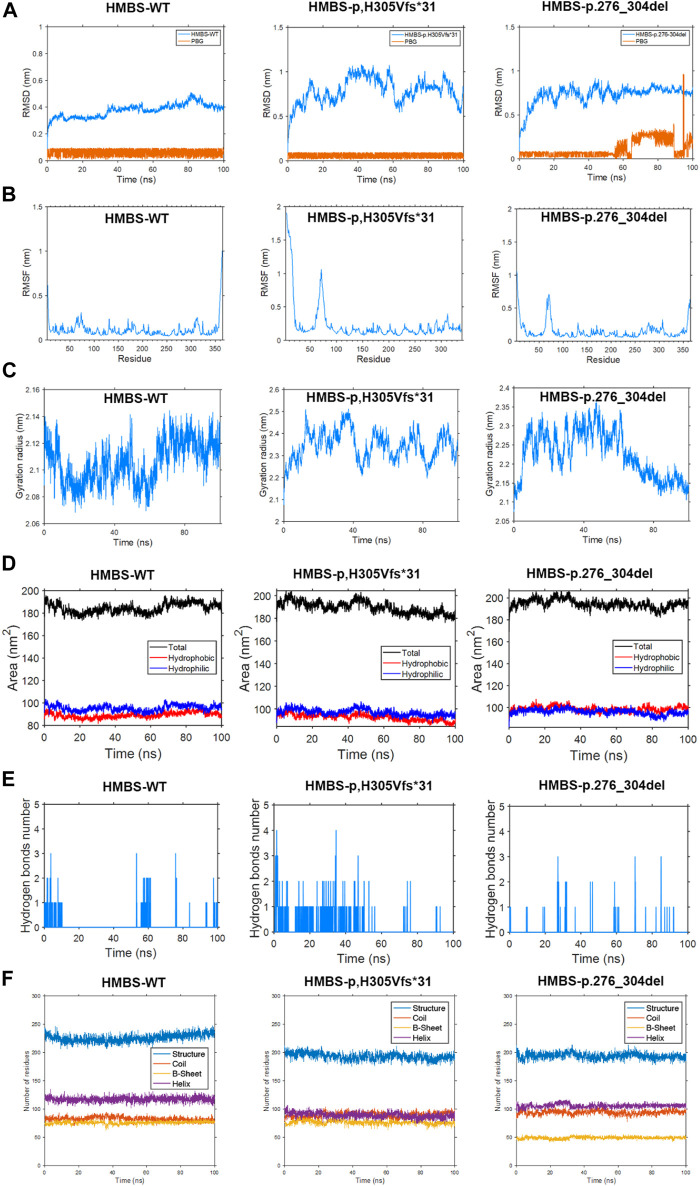
This study employs molecular dynamics simulation to investigate the *HMBS*-WT, *HMBS*-p.H305Vfs*31, and *HMBS*-p.276_304del examining differences in root mean square deviation, root mean square fluctuation, radius of gyration, solvent accessible surface, hydrogen bonding, and secondary structure. **(A)** Root mean square deviation difference. **(B)** Root mean square fluctuation difference. **(C)** Difference in radius of gyration. **(D)** Solvent accessible surface difference. **(E)** Hydrogen bond difference. **(F)** Secondary structure difference.

According to the findings of the root mean square deviation (RMSD) analysis, it is evident that the p.H305Vfs*31 variant exhibits the most pronounced level of fluctuation, followed by the p.276_304del variant, while the WT demonstrates the least degree of fluctuation. Consequently, these observations suggest that the two mutations exert varying impacts on the conformation of the proteins (Figure 4A). The conformation of the WT protein achieves a steady state after 70 ns, while the PBG small molecule reaches a steady state after 20 ns. Similarly, the conformation of the p.H305Vfs*31 protein reaches a steady state after 70 ns, and the state of the PBG small molecule reaches a steady state after 60 ns. However, after 80 ns, the conformation of the p.276_304del protein reaches a steady state, but the state of the PBG small molecule remains unstable, suggesting that PBG does not bind to the protein (Figure 4A).

The amino acid residues of the WT, p.276_304del, and p.H305Vfs*31 exhibit similarity in the first 275 residues, thereby enabling a comparison of their root mean square fluctuations (RMSF). Upon analysis, it was observed that the WT displays greater fluctuations in the head, tail, and chain ends proximal to amino acids 70, 130, 170, 200, 275, and 310. Conversely, the p.H305Vfs*31 exhibits more erratic fluctuations in the head region and near amino acid 70. Additionally, the p.276_304del demonstrates increased erratic fluctuations in the head region, near amino acid 70, and in close proximity to the tail. In comparison to the WT, the p.H305Vfs*31 exhibits the most pronounced degree of head fluctuation, while its tail experiences the least fluctuation due to truncation. Additionally, the p.276_304del variant’s tail demonstrates reduced fluctuation compared to the WT, likely attributable to the incurred damage. Consequently, it can be inferred that the residue conformation of the head, specifically at positions 70 and 250–270 in both the p.276_304del and p.H305Vfs*31 variants, exerts significant effects (Figure 4B).

The measurement of protein conformation compactness was conducted using the radius of gyration (Rog). Analysis of the results reveals that the radius of gyration remains constant for both the WT and p.H305Vfs*31 after 70 ns, suggesting that their conformations have reached a stable state. Similarly, the radius of gyration for the p.276_304del remains unchanged after 80 ns, indicating the attainment of a steady conformation. Upon comparing the outcomes of the three analyses, it becomes evident that WT exhibits the smallest Rog, thereby signifying its superior compactness. Conversely, p.H305Vfs*31 displays the largest Rog and the most substantial fluctuation, thereby indicating a comparatively looser structure when compared to both WT and p.276_304del (Figure 4C).

The solvent accessible surface area (SASA) refers to the surface area of a protein that is in contact with a solvent. Typically, a higher SASA value indicates a greater contact area between the protein and the solvent, resulting in increased exposure of the internal structure. The results of the analysis of the SASA values obtained from a 100 ns molecular dynamics simulation indicate that the WT exhibited a hydrophilic surface area of 94.42 nm^2^ and a hydrophobic surface area of 87.58 nm^2^. In contrast, the p.H305Vfs*31 displayed a hydrophilic surface area of 95.52 nm^2^ and a hydrophobic surface area of 89.87 nm^2^. Lastly, the p.276_304del resulted in a hydrophilic surface area of 94.27 nm^2^ and a hydrophobic surface area of 100.27 nm^2^. The p.H305Vfs*31 exhibited the greatest degree of hydrophilicity, while the p.276_304del displayed the highest level of hydrophobicity. These findings suggest that the internal structural domains of both variants were more exposed compared to the WT, potentially influencing their functional characteristics (Figure 4D).

The analysis results of hydrogen bond differences indicate that the WT protein is both compact and undamaged, exhibiting a significantly greater number of hydrogen bonds in comparison to the p.276_304del and p.H305Vfs*31. The reduction in internal hydrogen bonds within the p.276_304del and p.H305Vfs*31 negatively impacts their structural integrity and compactness, potentially exacerbating functional issues. During the simulation, it was observed that the quantity of internal hydrogen bonds formed between the WT and PBG ranged from 1 to 2 pairs. Conversely, the number of internal hydrogen bonds between the p.H305Vfs*31 and PBG exhibited a gradual decline, ultimately reaching 0 pairs. This decline suggests a weakening interaction between the p.H305Vfs*31 and PBG, ultimately leading to the absence of any interaction. Similarly, the number of internal hydrogen bonds between the p.276_304del and PBG also gradually decreased to 0 pairs during the simulation, indicating a lack of interaction between the p.276_304del and PBG (Figure 4E).

Based on the analysis of secondary structure, the alterations induced by p.276_304del and p.H305Vfs*31 are primarily reflected in the composition of protein secondary structures. In comparison to p.276_304del and p.H305Vfs*31, the WT exhibits a considerably greater content of secondary structure. Notably, p.276_304del displays the lowest amount of secondary structure due to its limited 200 amino acids within the secondary structure. In comparison to the WT protein, which possesses 120 functional residues capable of forming α-helices, the p.H305Vfs*31 only contain 85 α-helices. Furthermore, variations in the composition of other secondary structures are also observed among the WT, p.276_304del, and p.H305Vfs*31 proteins. It is plausible that the alterations in p.276_304del and p.H305Vfs*31 result in modifications to the protein’s secondary structure, potentially leading to functional disparities (Figure 4F).

### 3.5 Modeling mutations induced abnormalities in enzyme activity of the HMBS enzyme through molecular interactions

**FIGURE 5:**
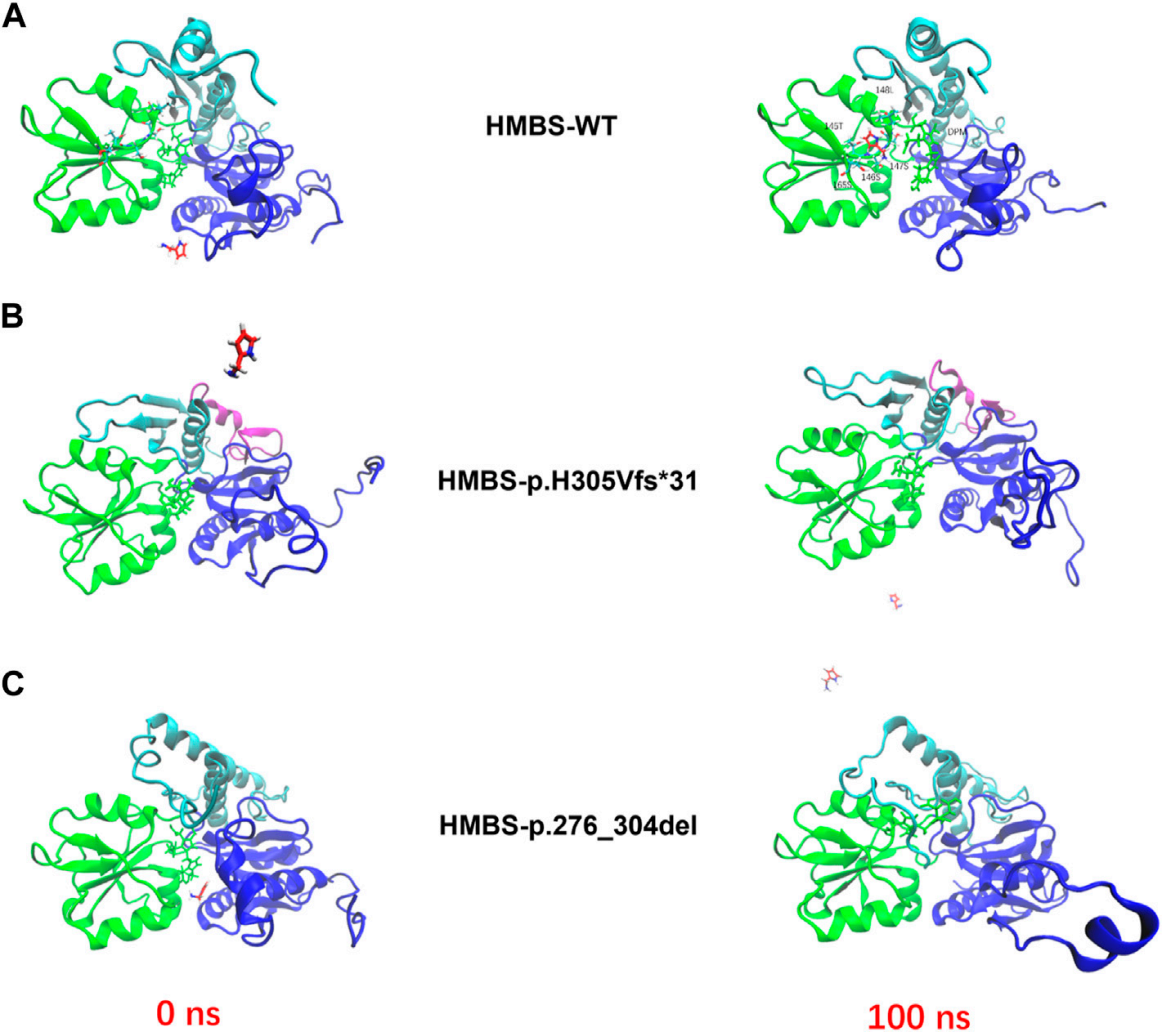
The enzyme activity simulations of *HMBS*-WT, *HMBS*-p.H305Vfs*31, and *HMBS*-p.276_304del were conducted using molecular docking. **(A)** *HMBS*-WT enzyme activity simulation based on molecular docking. **(B)** *HMBS*-p.H305Vfs*31 enzyme activity simulation based on molecular docking. **(C)** *HMBS*-p.276_304del enzyme activity simulation based on molecular docking. The red and green licorices represent for the outcoming PBG molecule and inside DPM active site along the protein backbone, respectively.

Based on the outcomes of homologous modeling subsequent to molecular dynamics analysis, it has been observed that p.276_304del and p.H305Vfs*31 exhibit substantial alterations in numerous regions when compared to the wild type. The employment of molecular dynamics technique facilitates the representation of atomic details by allowing simulated proteins to undergo conformational motion over time. Following molecular dynamics analysis, homologous modeling is executed to achieve a conformational transformation of simulated mutant proteins that is more closely aligned with the actual scenario (Figures 5A–C). The binding pocket/active site represent for the *HMBS* crystal structure (PDB ID: 3ECR) with the DPM cofactor in the centre of the protein. The binding interaction is a stepwise condensation, which starts from a DPM cofactor in the protein, creating enzyme intermediate complexes bound to one, two or three PBG molecules. In the simulation, we focus on the binding process of one protein to one PBG molecule. PBG goes from outside to active region in wild type protein through the 145T, 146S, 147S, 148L, 165S amino acid and DPM center interactions between the PBG small molecule and the protein (Figure 5A).

Mutations have been observed to induce alterations in the structure of *HMBS* proteins. To ascertain the enzymatic activity of the mutant proteins p.276_304del and p.H305Vfs*31, we employed computer simulations to investigate their interactions with PBG small molecules. Our findings indicate that the WT protein exhibited robust interactions with PBG small molecules, with the binding site situated in close proximity to the active center of DPM (Figure 5A). However, PBG small molecules exhibit a considerable distance from the DPM active centers of p.276_304del and p.H305Vfs*31, and demonstrate a limited ability to interact with either (Figures 5B, C). According to our research, it is probable that the two mutant proteins exhibit a near-complete state of inactivity.

## 4 Discussion

In our study, we discovered a previously unknown mutation that impacts the splicing of the *HMBS* gene in a female patient with AIP and her daughter. The mutation is found at the intersection of exon 13 and intron 13. In most human genes, the coding regions are discontinuous and contain multiple exons. After transcription of the gene, mRNA precursors are produced. These precursors undergo splicing, during which introns are removed and exons are joined together to form functional gene molecules. To initiate the splicing reaction, the Splicingosome must recognize the Splicing site at the exon-intron junction, which includes the 5′ Splicing donor located in the intron and the 3′ Splicing acceptor located in the intron (Maquat, 1996; Hastings and Krainer, 2001). The majority of introns have GT and AG boundary sequences that are essential for Splicing recognition (Burset et al., 2000; Patel and Steitz, 2003). Our study revealed that the mutation c.912 + 1G>C impaired the 5′Splicing donor in intron 13 by damaging its 5′Splicing. This led to an incorrect recognition of the GT position of exon 13, resulting in the cleavage of 87 nucleotides at the end of exon 13. Due to a misreading of the GT position of exon 13, 87 nucleotides were cleaved by the Splicing, resulting in a *HMBS* protein lacking amino acids 276-304. Another discovery made in the study was that c.912 + 1G>C disrupts Splicing recognition, leading to the production of a *HMBS* protein that retains intron 13. The *HMBS* truncated protein is composed of 304 normal amino acids and 30 abnormal amino acids due to a premature termination codon tag that was inserted at nucleotide 88 downstream of exon 13 of the *HMBS* gene. This mutation causes the protein synthesis to end prematurely, resulting in a truncated protein that may affect its folding and stability. The *HMBS* gene mutation Trp198Term is the most common in Swedish patients. This mutation results in severely truncated, possibly inactive, and easily degradable proteins. Patients with this mutation have the highest incidence rate of AIP. Our study found that p.H305Vfs*31 causes the *HMBS* protein to shift from the 305th amino acid, resulting in early translation termination. This sequence change in domain 3 of p.H305Vfs*31 exceeds 40%, which interferes with enzyme folding during biosynthesis and destroys its stability. Our observations indicate that the splicing of *HMBS* gene mRNA in the peripheral blood of patient is normal, with stronger normal subtype bands compared to the abnormal subtype bands. This suggests that the WT-*HMBS* protein plays a dominant role in patient, which may indicate that the symptoms are not severe.

The *HMBS* protein is divided into three domains. Domain 1 is discontinuous and comprises residues 1–116 and 216–239. Domain 2 is made up of residues 117–215, while domain 3 is composed of residues 240–361 (Song et al., 2009). The three domains are connected through the flexure hinge region. The interface between domains 1 and 2 is where the active site of DPM cofactor is located (Bung et al., 2018). The formation of porphyrin is catalyzed by combining *HMBS* with DPM cofactor in the same catalytic position, which results in four repeated reactions. Domain 3 of the protein consists of an antiparallel β-sheet with three chains, one of which is covered by three α-helices. This domain interacts equally with domains 1 and 2, thereby contributing to the overall stability of the protein (Gill et al., 2009). Compared to *Escherichia coli* (*EcHMBS*) and *Arabidopsis thaliana* (*AtHMBS*), *hHMBS* has a 29-residue segment (residues 296–324) located at the interface between domain 1 and 3. The functions of the 29-residue inserts, which are located far from the active site, are still unknown. However, they are positioned between domain 1 and domain 3, which suggests that they may influence the interaction between domain 2 and domain 3 by pushing domain 3 towards domain 2. This could explain why *hHMBS* has a lesser movement of the domain compared to *EcHMBS*, possibly due to the presence of the 29-residue inserts (Bung et al., 2018). Furthermore, it is postulated that the interplay between the 29-residue segment and domain 1 may modulate conformational fluctuations that are pertinent to enzyme interactions, leading to feeble dimerization of the crystal. Our investigation reveals that the impairments in domain 3 of p.276_304del and p.H305Vfs*31 compromise the integrity of the 29-residue region of *hHMBS*, thereby impacting the overall stability of the enzyme. In light of these findings, it is hypothesized that the aberrations in residues 296–324 of p.276_304del and p.H305Vfs*31 may indirectly influence the rate of porphyrinogen formation catalyzed by *hHMBS*.

Polypyrrole extension can be achieved by catalyzing with *HMBS* using the same catalytic site four times. The timely transfer of the reaction intermediate from the active site is crucial, and it is closely linked to the flexible sliding ring E250-C261 (Pluta et al., 2018). Our study highlights the importance of the flexible sliding ring, which is crucial for reaction extension. The slip ring comprises of seven residues (E250, A252, L254, H256, E258, G260, and C261), and any mutations in these residues can result in AIP. This can cause a decrease in the fluidity of the ring, leading to reduced reaction efficiency. Our RMSF analysis has revealed that residue fluctuations in p.276_304del and p.H305Vfs*31 in the 250–270 region are significantly higher than in WT, indicating their potential role in affecting the ring’s flexibility. There is a possibility that the reduced efficiency of the reaction in p.276_304del and p.H305Vfs*31 could be due to damage to the 29-residue insert. This damage may cause a reduction in the insert’s restrictions on the movement of domain 3, resulting in an increased degree of freedom for flexible sliding ring. Additionally, the RMSF analysis of the active site loops (residues 56–76) of WT, p.276_304del, and p.H305Vfs*31 showed significant differences between them. The RMSF values of p.276_304del and p.H305Vfs*31 were significantly lower than those of WT, with the greatest difference observed between p.H305Vfs*31 and WT. Based on our analysis, it is possible that further research could support our findings. Specifically, when the 29-residue insert is damaged, the flexible sliding ring has more freedom to react during polypyrrole extension. This reduced efficiency of the active site loops to respond ultimately leads to a decrease in enzyme activity.

In addition to serving as the main connection between domain 1 and domain 2, the 29-residue in domain 3 also plays a role in filling domain 1. This interaction is primarily facilitated through hydrogen bonds (Gill et al., 2009). The residues 312–315 and 318–321 create antiparallel chains. Upon bifurcation of the chain, the hydrogen bonds between Leu315NH-Ile318CO and Leu315CO-Ile318NH in the main chain are broken and replaced by the main chain-side chain interaction Asp312CO-Arg321NH (Gill et al., 2009). The side chain of Arg251 is involved in a hydrogen bond with the carbonyl oxygen of Val316. The hydrophobic side chains of 29-residues, namely, Leu315, Val316, Ile318, and Ala320, are observed to interact with Leu329, Ile248, and Leu244 of domain 3. In addition, the interaction between domains 1 and 3 is stabilized through the formation of hydrogen bonds involving Gly317CO-Ile110NH and Thr319NH-Ile110CO, as well as the hydrogen bonds formed between the side chain of Gln314 and the carbonyl group of Phe108, and the OG atoms of Thr109 and Thr319 (Gill et al., 2009). The present study reveals that domain 3 of p.H305Vfs*31 exhibits complete aberrance from the 305th residue, thereby suggesting that the interaction between domains 1 and 3 of p.H305Vfs*31 is considerably weaker than that observed in the WT.

Hydrophobic interactions occur between certain residues within the three domains of *HMBS* (Gill et al., 2009). For instance, the residues Leu97, Leu244, and Cys247 are situated amidst domains 1 and 3, while the residues Ala152, Met212, and Leu285 are located between domains 2 and 3. Additionally, the residues Trp283 and Gln153 are positioned between domains 2 and 3 (Gill et al., 2009). In our study, we found that p.276_304del lacks key sites, specifically Leu285 and Trp283, which play a crucial role in maintaining domain 3 interactions. As a result, p.276_304del has reduced hydrophobicity compared to the wild type, making it more difficult for the protein to fold inward and form secondary structures, as well as domains and tertiary structures. The decrease in hydrophobicity does not contribute to the formation of α-helix and this negatively affects the stability of the protein. Moreover, the conservative hydrophobic side chain Leu245 in domain 3 interacts with Pro241, Pro302, and Pro327 in domain 1. The substitution of Leu245 with Arg245, as seen in AIP, results in the burial of a large positively charged side chain, which in turn affects the folding stability of domain 3 (Gill et al., 2009). The findings of our study indicate that the absence of Pro302 in p.276_304del and Pro327 in p.H305Vfs*31, as well as the absence of the crucial interaction sites that bind with Leu245, have a detrimental impact on the folding stability of domain 3.

The WT exhibits 80 random coil structures, 78 β-sheet structures, and 120 α-helix structures. The p.H305Vfs*31 exhibits 90 random coil structures, 78 β-sheet structures, and 85 α-helix structures. Similarly, the p.276_304del displays 95 random coil structures, 50 β-sheet structures, and 105 α-helix structures. The p.276_ 304del exhibits the lowest β-sheet structure, while the p.H305Vfs*31 displays the lowest α-helix structure. Conversely, the WT demonstrates the highest α-helix structure. The interaction between PBG small molecules and the WT is robust, with the binding site accurately positioned near the active center of DPM. In contrast, the affinity between PBG small molecules and p.276_304del and p.H305Vfs*31 exhibited relatively low levels of interaction. The c.912 + 1G>C mutation results in the production of p.276_304del and p.H305Vfs*31, which contain defective amino acid regions, in the 29-residue insert located distally from the active center. Our computer simulations indicate that the PBG small molecule cannot bind to the DPM active centers of these defective regions, preventing the catalytic reaction from proceeding. Thus, we predict that p.276_304del and p.H305Vfs*31 will lose their catalytic activity completely. Prior to our findings, the role of the 29-residue insert region was unclear. Our research shows that this region is crucial in maintaining the conformational stability of the *hHMBS* protein. Failure of this region can lead to a complete loss of enzyme activity.

AIP can be diagnosed clinically by the elevation of urinary ALA and PBG, but these levels may not always be elevated in asymptomatic patients. Hence, genetic testing is crucial in diagnosing AIP in asymptomatic patients. The *HMBS* gene mutation shows a high level of genetic diversity and familial transmission. When conducting family screening for AIP patients, there is a good chance that other family members may have the same mutation site. Advising potential AIP patients or their guardians to avoid various inducements of AIP can help reduce acute attacks. Following the ACMG guidelines, we rated the *HMBS*: c.912 + 1G>C mutation as “pathogenic”. The following evidence substantiates the pathogenicity rating. PVS1_Strong: functional deletion is a known pathogenic mechanism resulting from *HMBS* gene abnormalities. The c.912 + 1G>C variation, a classical Splicing site variation, is also associated with functional loss of the gene. PM2_Supporting: the variation is infrequent and not included in the gnomAD database for the East Asian general population. PM4: the length of the protein is modified by the incorporation of a non-repetitive region. PP3: The bioinformatics software predicts that variation has an impact on splicing. PP4: The family history and phenotype of the individual carrying the mutation exhibit a high degree of consistency with AIP.

## 5 Conclusion

In summary, our study has identified and confirmed the existence of a novel *HMBS* splicing variant, namely, c.912 + 1G>C (p.276_304del and p.H305Vfs*31). Furthermore, the ClinVar database currently designates the NM_000190: c.912 + 1G>C variant as a “pathogenic” alteration. Our experimental findings provide robust evidence to support the pathogenicity of this variant.

## Data Availability

The original contributions presented in the study are included in the article/Supplementary Materials, further inquiries can be directed to the corresponding author.

## Abbreviations

1000G, 1000 Genome Project; ACMG, American College of Medical Genetics and Genomics; AIP, Acute intermittent porphyria; ALA, 5‘-aminolevulinic acid; ALAS1, 5′-aminolevulinate synthetase 1; ClinGen, Clinical Genome Resource; dbSNP, Database of single nucleotide polymorphisms; DPM, Dipyrromethene; ExAC, Exome Aggregation Consortium; gnomAD, Genome Aggregation Database; GRCh37, Genome References Consortium Human Build 37; HMBS, Hydroxymethylbilane synthetase; NPT, Constant-pressure, constant-temperature; NVT, Canonical ensemble; PBG, porphobilinogen; PDB, Protein Data Bank; RMSD, Root mean square deviation; RMSF, Root mean square fluctuation; Rog, Radius of gyration; SASA, Solvent accessible surface area; WT, Wild-type.
